# Oral Biofilms from Symbiotic to Pathogenic Interactions and Associated Disease –Connection of Periodontitis and Rheumatic Arthritis by Peptidylarginine Deiminase

**DOI:** 10.3389/fmicb.2018.00053

**Published:** 2018-01-30

**Authors:** Katja Kriebel, Cathleen Hieke, Brigitte Müller-Hilke, Masanobu Nakata, Bernd Kreikemeyer

**Affiliations:** ^1^Institute of Medical Microbiology, Virology and Hygiene, University of Rostock, Rostock, Germany; ^2^Institute of Immunology, University of Rostock, Rostock, Germany; ^3^Department of Oral and Molecular Microbiology, Osaka University Graduate School of Dentistry, Suita-Osaka, Japan

**Keywords:** oral biofilm, periodontitis, rheumatic arthritis, *P. gingivalis*, peptidylarginine deiminase

## Abstract

A wide range of bacterial species are harbored in the oral cavity, with the resulting complex network of interactions between the microbiome and host contributing to physiological as well as pathological conditions at both local and systemic levels. Bacterial communities inhabit the oral cavity as primary niches in a symbiotic manner and form dental biofilm in a stepwise process. However, excessive formation of biofilm in combination with a corresponding deregulated immune response leads to intra-oral diseases, such as dental caries, gingivitis, and periodontitis. Moreover, oral commensal bacteria, which are classified as so-called “pathobionts” according to a now widely accepted terminology, were recently shown to be present in extra-oral lesions with distinct bacterial species found to be involved in the onset of various pathophysiological conditions, including cancer, atherosclerosis, chronic infective endocarditis, and rheumatoid arthritis. The present review focuses on oral pathobionts as commensal and healthy members of oral biofilms that can turn into initiators of disease. We will shed light on the processes involved in dental biofilm formation and also provide an overview of the interactions of *P. gingivalis*, as one of the most prominent oral pathobionts, with host cells, including epithelial cells, phagocytes, and dental stem cells present in dental tissues. Notably, a previously unknown interaction of *P. gingivalis* bacteria with human stem cells that has impact on human immune response is discussed. In addition to this very specific interaction, the present review summarizes current knowledge regarding the immunomodulatory effect of *P. gingivalis* and other oral pathobionts, members of the oral microbiome, that pave the way for systemic and chronic diseases, thereby showing a link between periodontitis and rheumatoid arthritis.

## Introduction

**FIGURE 1:**
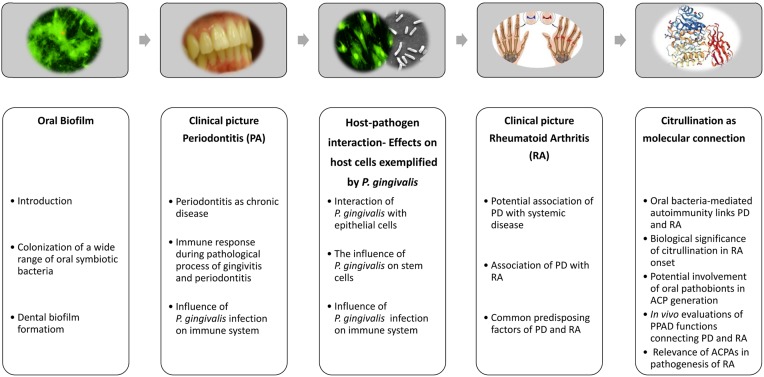
Brief overview of current concepts regarding onset of periodontitis and rheumatic arthritis, and deduced causal relationships between both diseases. After establishing a subgingival biofilm, oral pathobionts, including *Porphyromonas gingivalis*, induce periodontitis as a chronic disease, which is attributable to host-pathobiont interactions and deleterious host immune responses in periodontal tissues. Dysregulated citrullination caused by the pathobiont *Porphyromonas gingivalis* has been suspected to be a causative factor for onset of rheumatic arthritis. Parts of this figure were taken from freely available web resources: https://www.chirurgie-portal.de/innere-medizin/rheuma.html; www.rcsb.org/pdb/ngl/ngl.do?pdbid=5AK7 (Rosenstein and Hildebrand, 2015; Montgomery et al., 2016).

The oral cavity is a unique habitat that allows for colonization of a wide variety of commensal microbial species, as it supplies a diversified nutrient influx as well as high humidity and variable oxygen concentrations. Furthermore, the existence of soft (gingiva) and non-shedding hard (teeth) tissues provides microorganisms with potential surfaces for adherence and subsequent interaction with various host cells. Colonization of the oral cavity in healthy individuals is based on balanced bacteria-host and interbacterial interactions. The continuous existence of dental plaques in gingival tissues and interactions of pathobionts with host cells cause inflammation, leading to periodontitis (PD). There is also increasing evidence suggesting an association of chronic PD with other types of systemic inflammatory diseases, such as atherosclerosis, infective endocarditis, diabetes, adverse pregnancy outcome, respiratory diseases, and rheumatoid arthritis (RA) (Li et al., 2000; Pihlstrom et al., 2005; Kim and Amar, 2006; Gaffen et al., 2014; Hajishengallis, 2015). This raises the question whether the periodontal microbiota is bystander or responsible for the initial step of chronic diseases. In the present review, the pathogenic mechanism of PD is introduced from the perspective of host bacteria/interbacterial interactions and host immune responses. Moreover, interactions of the pathobiont *Porphyromonas gingivalis* with host cells, as well as a possible link between the pathobiont and RA are discussed (**Figure 1**).

## Colonization of Wide Range of Oral Symbiotic Bacteria

Initial bacterial host colonization occurs at birth, with *Staphylococcus epidermidis* and *Streptococcus* species detected within hours after birth (Nelson-Filho et al., 2013). The oral pioneer species *Streptococcus salivarius*, which has been detected within 8 h after birth (Rotimi and Duerden, 1981), represents the majority of oral bacteria with up to 98% of examined subjects showing its possession at the first tooth eruption (Cortelli et al., 2008). Dental structures and alterations in nutrition allow for further colonization of other bacterial species. Finally, the matured oral microbiome consists of hundreds of bacterial species, contributing to a complex community (Aas et al., 2005; Dewhirst et al., 2010). Gram-positive facultative anaerobic bacteria, such as the *Streptococcus* and *Actinomyces* genera, are predominant in healthy individuals, in whom a proper equilibrium between the oral microbiome and host immune responses is maintained with no signs of inflammation observed in the periodontium (Li et al., 2004; Jiao et al., 2014).

## Dental Biofilm Formation

After tooth surfaces are cleaned, their immersion in the fluid environment of the oral cavity causes surface adsorption of a thin acquired pellicle, which is mainly composed of saliva glycoproteins, such as proline-rich proteins, α-amylase, statherin, mucins, and agglutinin (Heller et al., 2017). Coating of those solid surfaces with a pellicle leads to changes in surface charge and free energy, thus promoting bacterial adhesion (Weerkamp et al., 1988). Bacteria attach to tooth surfaces in a diverse manner, ranging from specific interactions between pellicle components and bacterial surface molecules to charge-mediated weak interactions (Nesbitt et al., 1992; Jenkinson, 1994; Oli et al., 2006; Kolenbrander et al., 2010). The predominant initial colonizers of teeth are Gram-positive facultative anaerobic cocci and rods, including *Streptococcus* and *Actinomyces* species. These initial colonizers provide a foundation for further development of dental biofilm. *Streptococcus* recognizes components in the pellicle, such as a specific interaction between a pilus protein of *S. sanguinis* and salivary α-amylase (Okahashi et al., 2011). *Actinomyces* binds to proline-rich proteins and statherin, a phosphate-containing protein (Li et al., 2001). Once the initial colonizers attach to the surface, a biofilm mass develops through continued growth and subsequent adsorption of other bacterial species via coaggregation.

The surface molecules of these early colonizers allow for coaggregation of Gram-negative bacteria possessing a lower level of adherence to the pellicle, including members of the genera *Veillonella* and *Fusobacterium*. Bacteria belonging to the genus *Fusobacterium*, such as *Fusobacterium nucleatum*, are able to coaggregate with both initial and late colonizers, thus are called bridge species and known to promote successful development of dental biofilm. For bridging neighboring bacteria, *F. nucleatum* utilizes surface molecules such as RadD, an arginine-inhibitable adhesin, and the fusobacterial apoptosis protein Fap2 (Kaplan et al., 2010). Habitat analysis of the oral microbiome has suggested that the genus *Corynebacterium* is strikingly specific for supragingival and subgingival plaque, with *Corynebacterium matruchotii* dominant among six species deposited in the Human Oral Microbiome Database (Dewhirst et al., 2010). Since this genus has been found in only trace amounts in saliva and other specimens from different anatomical sites, it is considered to have a specific role in dental biofilm formation. In fact, in a study that utilized combinational labeling and spectral imaging FISH (CLASI-FISH), Mark Welch et al. (2016) observed a complex microbial consortium, termed a hedgehog structure, mainly consisting of nine taxa arranged in an organized spatial framework, including *Corynebacterium, Streptococcus, Porphyromonas, Haemophilus*/*Aggregatibacter, Neisseriaceae, Fusobacterium, Leptotrichia, Capnocytophaga*, and *Actinomyces*. This plaque hedgehog represents a radially organized structure, of which the main framework is primarily composed of *Corynebacterium* with a multi-taxon filament-rich annulus and peripheral corncob structures. In the corn-cob structures, *Corynebacterium* filaments are surrounded primarily by *Streptococcus*, though *Porphyromonas* and *Haemophilus*/*Aggregatibacter* are also in close contact with streptococcal cells, while the filament-rich annulus is mainly composed of *Fusobacterium, Leptotrichia*, and *Capnocytophaga*. Thus, *Corynebacterium* organisms are considered to be bridge-species in regard to biofilm formation. Bridge-species further coaggregate late colonizers that have effects on PD pathogenesis.

Each anatomical site in the oral cavity possesses a distinct composition of biofilm members that affects the local environment by intrinsic metabolism. Stratification and selective interaction between distinct bacterial species in dental biofilms are conducted by mutually antagonistic and cooperative interactions, which are attributable to environmental/metabolite gradients and quorum sensing (Brown and Whiteley, 2007; Ramsey et al., 2011; Zhu and Kreth, 2012; Wessel et al., 2014). Tooth-related plaque biofilm can be generally classified based on location into supragingival, formed above the gingival margin, and subgingival, formed below the gingival margin. When a pathological dental pocket becomes formed between a tooth surface and gingiva during the course of PD onset, an anaerobic condition is built up. Moreover, major sources of nutrition for subgingival plaque bacteria are provided via inflammatory periodontal tissues and gingival cervical fluid originating in blood, since permeation of saliva components is more or less limited. Consequentially, subgingival plaque in the pocket is dominated by anaerobic and motile bacteria as compared with supragingival plaque, as noted in detail below. Interactions of obligate anaerobic bacteria, such as *P. gingivalis*, with host cells have been implicated in the pathogenesis of PD.

## Periodontitis as Chronic Disease

**FIGURE 2:**
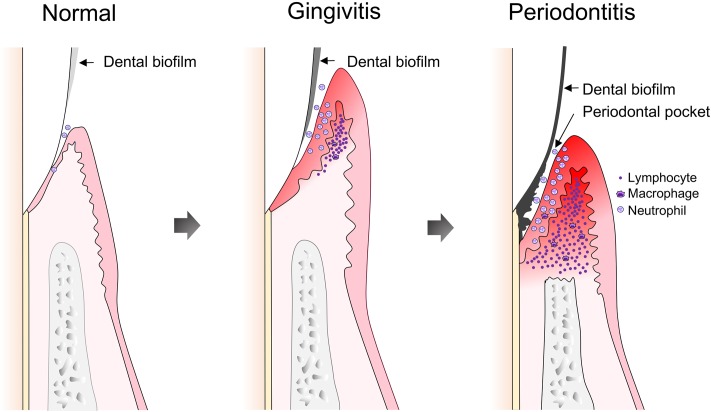
Development of gingivitis and periodontitis. Following dental plaque accumulation, neutrophils dominate the host immune response, accompanied by progression of an early or stable gingivitis lesion, along with increased infiltration of macrophages and T cells. The gingivitis lesion develops into a periodontitis lesion, which is characterized by formation of a pathogenic periodontal pocket and destruction of periodontal tissues. Infiltrated lymphocytes are dominated by B and plasma cells.

Establishment and maturation of periodontal dental biofilms are characterized by co-aggregation of opportunistic microorganisms caused by diverse factors, including poor oral hygiene. Infection of periodontal host cells as well as expression of virulence factors can provoke a local inflammatory response. Initial periodontal tissue inflammation is termed gingivitis and its pathology can be resolved by removal of dental biofilms (**Figure 2**). On the other hand, continuous existence of stable plaques, including accumulation of opportunistic bacterial species, supports long-lasting inflammation. A shift in the periodontal microbiome that accompanies an increase in Gram-negative anaerobic species is now accepted as an indicator of periodontal disease (Yano-Higuchi et al., 2000; Klein and Goncalves, 2003; Yang et al., 2004; Berezow and Darveau, 2011; He et al., 2015). Such a shift in composition affects host immune responses, and leads to dysbiosis between the oral microbiota and the host (Hajishengallis and Lamont, 2012). Therefore, following establishment of gingivitis, PD develops as a chronic inflammatory condition.

Periodontitis is characterized by irreversible and progressive degradation of periodontal tissues. With continuous inflammation, proliferation of epithelial cells connecting tooth surfaces and gingival tissues causes detachment of the cell layer, and subsequent formation of a pathogenic dental pocket between teeth and gingival tissues (**Figure 2**). The resulting micro-environment is characterized by reduced oxygen concentration or even anoxic areas. Mettraux et al. (1984) quantified oxygen concentrations in the periodontal pockets of patients with untreated PD and found them to range from 0.7 to 3.5%. On the other hand, the progression and severity of PD are strongly dependent on the quality and quantity of microorganisms harbored in periodontal plaque, as well as individual risk factors, e.g., age, genetic predisposition, systemic disorders, and lifestyle aspects, such as dental hygiene, nutrition, and smoking (Pihlstrom et al., 2005; van Dyke and Sheilesh, 2005; Hajishengallis and Lamont, 2012; Heaton and Dietrich, 2012; AlJehani, 2014).

Approximately 90% of microorganisms isolated from periodontal pockets are strictly anaerobic (Slots, 1977; Uematsu and Hoshino, 1992) and certain sets of bacteria have been frequently detected at elevated levels in periodontal lesions as compared with healthy tissues. Socransky et al. (1998) analyzed distribution of approximately 40 species in subgingival plaque using a DNA–DNA hybridization technique. Findings from DNA cluster analysis indicated that typical co-colonization of specific oral species, among which a cluster with the nomenclature “red complex” composed of the Gram-negative anaerobic species *Tannerella forsythia, P. gingivalis*, and *Treponema denticola*, is associated with increased pocket depth and bleeding upon clinical pocket probing, while the other four clusters examined were not shown to be associated with clinical parameters indicating periodontal disease. This pattern of oral colonization was also confirmed to exist in supragingival plaque samples (Haffajee et al., 2008). Aas et al. (2005) also identified the three bacterial species of the red complex as highly associated with disease status, which confirmed the colonization model by Socransky et al. (1998), and those findings were later supported by other studies (Dewhirst et al., 2010; Zarco et al., 2012; Wade, 2013; Duran-Pinedo and Frias-Lopez, 2015). Presently, the association of particular bacterial species within an intricate microbial community with periodontal health status is widely accepted.

Accumulation of opportunistic bacteria in periodontal plaques and their deleterious effects on host tissues via specific virulence factors provoke host immune responses. In response to microbial challenge, a massive cytokine response occurs, which triggers activation and recruitment of polymorphonuclear leukocytes (PMNs) in periodontal pockets (**Figure 2**). Their activation and oxidative burst contribute to periodontal homeostasis damage and subsequent degradation of periodontal tissues (Waddington et al., 2000; Kantarci et al., 2003; Graves, 2008). As compared to healthy individuals, the number of PMNs is increased in both periodontal pockets and the bloodstream of patients with chronic PD (Lakschevitz et al., 2013; Kolte et al., 2014), thus sustaining inflammation.

## Immune Response During Pathological Process of Gingivitis and Periodontitis

The concept of chronic PD as an immunological disease, which was proposed more than 40 years ago (Seymour et al., 1979), implies that a primary etiologic factor is bacterial infection that elicits a specific immune response by the host, triggering gingival inflammation and progression to chronic PD. Over the past 20 years, a number of studies have investigated and defined immune system components contributing to its pathogenesis. For example, it has been shown that both innate and adaptive immune systems are involved in PD onset, in which the roles of T- and B-lymphocytes are likely to be equally crucial (Gonzales, 2015). However, in regard to polarization of T-helper (Th) cell response, it remains elusive whether PD pathogenesis is driven by Th1, Th2, or Th17, or what role is adopted by regulatory T cells (Tregs) (Carvalho-Filho et al., 2016).

A plausible model for the pathological process of PD has been suggested, based on histopathological examinations of PD tissue sections. A pathological condition develops in sequential order and PD progression is subdivided into various stages, starting with initial lesion formation during the first 4 days after plaque accumulation. PMNs, i.e., neutrophils, dominate the host immune response, accompanied by the activation of complement component C3 via an alternative pathway. Subsequent production of the anaphylatoxins C3a and C5a leads to activation of mast cells with release of vasoactive substances that facilitate vascular permeability and development of edema. Moreover, mast cells release TNF-α, which up-regulates the expression of adhesion molecules on endothelial cells, allowing for increased PMN infiltration (Ohlrich et al., 2009). After approximately 4–7 days of plaque accumulation, the initial lesion progresses to an early or stable gingivitis lesion with increasing infiltration of macrophages and lymphocytes (**Figure 2**). Lymphocytes are predominantly T cells with a CD4-positive to CD8-positive ratio as high as 2:1, an activated phenotype that is at this point negative for the IL-2 receptor CD25. Since absence of CD25 indicates that T cells have proliferated elsewhere, characteristics of the early lesion indicate a delayed-type hypersensitivity reaction (DTH). The pathology can be stable for a certain period with equilibrium maintained between the immune system and microbiota, and inflammation confined to the gingiva. In cases when plaque is mechanically removed, the lesion will reversibly recover at this stage. However, if plaque accumulation is allowed to continue, and attachment between the gingiva epithelium and tooth surface is progressively lost, the stable lesion advances to an established or progressive PD lesion, characterized by a predominant response of B cells and plasma cells, high levels of IL-1 and IL-6, and periodontal tissue destruction, including alveolar bone loss (Ohlrich et al., 2009). The final stage, an advanced lesion, is also characterized by a dominance of B and plasma cells, while inflammatory status is exacerbated. Fibroblasts stimulated by IL-1β, TNF-α, and prostaglandin E2 secrete matrix metalloproteases (MMPs) that not only advance the lesion, but also accelerate bone loss (**Figure 2**). Palliative treatment of PD and complete removal of bacterial plaque improves the course of periodontitis and leads to arrest of the irreversible destruction of periodontal tissues.

Recent studies have shown the critical role of Th17 in maintenance of oral tissues. Individuals with a genetic defect in Th17 differentiation are susceptible to oral fungal infections (Liu et al., 2011; Moutsopoulos et al., 2015) and excess Th17 response in gingiva promotes inflammation, leading to deterioration related to periodontitis pathology (Eskan et al., 2012; Moutsopoulos et al., 2014). Dutzan et al. (2017) showed that the population of gingival IL-17-producing CD4+ T cells increases with age. Interestingly, Th17 responses are not dependent on colonization of commensal bacteria, which is totally different from those in the mucosa of other anatomical sites. Moreover, accumulation of gingival Th17 cells is dependent on physiological mechanical damage caused by mastication and subsequent induction of IL-6-mediated signals. Thus, mastication, a normal function of the oral cavity, shapes gingival immune homeostasis.

Even though the above sequence of events leading to chronic PD is feasible, it does not explain why the pathophysiological condition of an early lesion remains stable or even resolves in some individuals, while it progresses to B cell-driven progressive stages in others. The transition from a T cell- to B cell-rich lesion has been suggested to correlate with the transition from a Th1- to Th2-dominated response. Indeed, the pathological condition of chronic PD represents a pathology dominated by Th2 (Kinane and Bartold, 2007). Future research will be needed to investigate the involvement of Th17 and Treg cells, as well as the impact of environmental and genetic factors on susceptibility to chronic PD, and the underlying mechanisms for onset of PD-related systemic diseases.

## Host–Pathogen Interaction and Molecular Effects on Host Cells Exemplified by *P. gingivalis*

### Interaction of *P. gingivalis* with Epithelial Cells

The periodontal pathogen *P. gingivalis* infects gingival epithelial cells in the oral cavity, and its *in vitro* adherence to and internalization of epithelial cells have been well investigated. Dogan et al. (2000) observed invasion of primary epithelial cells by *P. gingivalis*, though it has been noted that the quantity of adherence and invasion of *P. gingivalis* are dependent on which human cell types and bacterial strains are investigated (Deshpande et al., 1998). For example, adherence rates of strain A7436 isolated from refractory PD to KB oral epithelial and human umbilical vein endothelial cells were found to be 1.1% and 0.5%, respectively (Deshpande et al., 1998). When comparing diverse *P. gingivalis* strains, the adherence rates vary, such as 0.5% for type strain W50 and 10.5% for strain 33277 to KB cells (Duncan et al., 1993; Dorn et al., 2000), while adhesion capacity also varies between cell types due to divergent interactions between *P. gingivalis* and the intrinsic cell surface. Furthermore, Saito et al. (2008) reported that strain ATCC 33 invaded Ca9-22 gingival epithelial cells as well as human aorta endothelial cells (HAEC) at higher rates as compared to strain W83, which might be explained by the highly fimbriated phenotype of strain ATCC33. Addition of *Fusobacterium nucleatum* strain TDC100 to that culture system increased the number of invaded bacteria for both strains (Saito et al., 2008). Pinnock et al. (2014) examined survival and bacterial release of *P. gingivalis* in a 3-D organotypic oral mucosal model, which was shown to mimic *in vivo* conditions. They found an increase of intracellular survival and bacterial release during incubation, as compared to monolayer experiments (Pinnock et al., 2014). These reports demonstrate that the interaction of epithelial cells with bacteria is dependent on a wide range of factors, while the high complexity of the oral cavity is not well represented by a monolayer cell culture system.

In addition to the composition of cells grown in various cell culture systems, host cell response itself is crucial for host signaling cascades. Toll-like receptors (TLRs) are pattern recognition receptors of epithelial cells as well as immune cells to recognize microbial molecules (Sugawara et al., 2006), which are involved in both intracellular and extracellular signaling pathways, culminating in activation of innate immune responses (Muzio and Mantovani, 2000). TLR2 and TLR4 respond to various bacterial factors, including lipoteichoic acid, lipopeptides, and lipopolysaccharide (LPS), and changes in their expression in gingival tissue during chronic PD have been demonstrated (Promsudthi et al., 2014). As an immunomodulation factor, *P. gingivalis* has effects on miRNA expression in host cells. miRNAs are single-stranded non-coding RNAs involved in regulatory processes, such as mRNA degradation and translational repression, and modulation of their expression results in dysregulation of proliferation and host cell immune responses (O’Connell et al., 2007; Aberdam et al., 2008). Gingival human oral keratinocytes incubated with heat-inactivated *P. gingivalis* exhibited up-regulation of miRNA-105, which is complementary to TLR2 mRNA (Benakanakere et al., 2009). Moreover, infection of primary gingival epithelial cells with viable *P. gingivalis* organisms was shown to significantly alter the expression of 14 miRNAs involved in regulation of apoptosis and cytokine secretion (Moffatt and Lamont, 2011). Overall, the oral cavity represents a complex network of numerous bacterial and/or host interactions, which can be disturbed by *P. gingivalis* via its utilization of epithelial cells to support its own survival.

### Influence of *P. gingivalis* on Stem Cells

The interactions of oral pathogens with differentiated cells, such as epithelial and bone cells, as well as stem cells and fibroblasts have been investigated. Stem cells can be isolated from various adult tissues, including bone marrow and gingiva (Barry and Murphy, 2004; Sonoyama et al., 2006; Tavian et al., 2006; Zhang et al., 2009; Jin et al., 2013). The source of human dental stem cells (hDSCs) is located in oral tissues and those exhibit the main characteristics of mesenchymal stem cells. hDSCs can be isolated from dental pulp and exfoliated deciduous teeth, as well as apical papilla, periodontal ligament, and dental follicle specimens (Gronthos et al., 2000; Miura et al., 2003; Seo et al., 2004; Jo et al., 2007; Sonoyama et al., 2008). The prominent presence of hDSCs in oral tissues provokes intriguing questions regarding whether *P. gingivalis* is able to interact with stem cells in tissues and, if so, what subsequent effects should be expected. The effect of the outer membrane component LPS of *P. gingivalis* on stem cells in regard to cell proliferation, viability, differentiating capacity, and immunomodulatory characteristics has been evaluated (Mysak et al., 2014; Chatzivasileiou et al., 2015; How et al., 2016), though the interaction of viable bacteria with stem cells remains poorly defined. Kriebel et al. (2013) demonstrated that stem cells and oral bacteria can be co-cultured under anaerobic conditions. In their system, oral microorganisms were less able to adhere to or internalize into human bone marrow stem cells (hBMSCs) in relation to gingival epithelial cells (Kriebel et al., 2013). Thereafter, additional studies revealed that human dental follicle stem cells (hDFSCs) elicit a reduced pro-inflammatory response following bacterial infection, as compared to differentiated cells (Biedermann et al., 2014). Furthermore, Kriebel et al. (2013) and Biedermann et al. (2014) showed that stem cell functions were influenced by oral bacteria *in vitro*, while Hieke et al. (2016) found that infection with viable bacteria induced distinct reactions by stem cells that were different from reactions to a single administration of LPS (Hieke et al., 2016). Thus, infected stem cells showed a reduced capacity for migration, though that finding is inconsistent with another study that demonstrated increased migration following stimulation with LPS (Chatzivasileiou et al., 2013). As compared with the analyses with LPS stimulation alone, data obtained in experiments with viable bacteria remain controversial. Additional studies are required to evaluate the reaction of stem cells to bacterial infection in human tissues.

Fibroblasts, the most predominant cell type in periodontal tissue, play important roles in tissue regeneration and PD-associated inflammation. They express TLRs, including TLR2 and 4 (Mahanonda et al., 2007), thus are considered to be involved in immune reactions to oral bacteria. The effects of *P. gingivalis* LPS on fibroblasts have been examined in regard to cell viability, immune response, and tissue repair, as well as the effects of cell signaling on those factors (Souza et al., 2010; Morandini et al., 2013; Sun et al., 2016). Furthermore, interactions of *P. gingivalis* with gingival fibroblasts have also been investigated, with the effects of immune modulating bacterial factors, the capsule, and gingipains, together with LPS, noted (O’Brien-Simpson et al., 2009; Brunner et al., 2010; Scheres and Crielaard, 2013). *P. gingivalis* can adhere to and invade human fibroblasts (Pathirana et al., 2008; Irshad et al., 2012; Zhang et al., 2014), and such bacterial infection induces secretion of pro-inflammatory mediators, including IL-6 and IL-8, via TLR-dependent and -independent pathways (Liu et al., 2014; Palm et al., 2015). In addition, *P. gingivalis* infection induces caspase-independent apoptosis as well as regulation of the inflammasome activation (Desta and Graves, 2007; Kuo et al., 2016). Such diverse responses of the infected fibroblasts affect biological functions and differentiation of other cell types, implicating their regulatory role in progression of periodontitis and consequent chronic inflammation (Zhang et al., 2014; Tzach-Nahman et al., 2017).

### Influence of *P. gingivalis* Infection on Immune System

The dental pocket is constantly exposed to oral microorganisms and innate immunity components permanently interact with bacteria. During inflammation, various cell types, including neutrophils and macrophages, migrate to the site of infection, with the former the first line of defense against invading microorganisms. In addition to phagocytosis, exocytosis of granules, release of reactive oxygen species (ROS), and induction of neutrophil extracellular traps (NETs) serve as anti-microbial factors (Segal, 2005). Interestingly, *P. gingivalis* is able to modify neutrophil activity in a manner that promotes neutrophil survival. It has also been shown that prolongation of neutrophil survival caused by *P. gingivalis* results in accumulation of neutrophils in adult patients with PD (Gamonal et al., 2003), while later it was reported that neutrophils isolated from the blood of chronic PD patients were highly reactive to stimulation by *P. gingivalis* LPS, with increased release of the pro-inflammatory cytokine IL-8 (Restaino et al., 2007). Furthermore, neutrophils from patients with localized aggressive PD produce higher levels of ROS against *P. gingivalis*, as compared with those from healthy donors, and release of ROS results in secretion of pro-inflammatory cytokines, which can be advantageous to counteract the increased burden of *P. gingivalis* (Damgaard et al., 2016). As a strategy for escape from the host immune system, it has been shown that *P. gingivalis* has an ability to invade epithelial cells. Moreover, triggering of an immune response can be beneficial for colonizing deeper tissues of the host (Li et al., 2008). The working group of Hajishengallis noted that direct interaction of *P. gingivalis* with PMNs resulted in modulation of the neutrophil killing function via MyD88, an adaptor protein of TLR2 and TLR4 receptors (Hajishengallis et al., 2008; Maekawa et al., 2014).

Macrophage functions are also modulated by *P. gingivalis*. Macrophage migration-inhibitory factor (MIF) is involved in killing of bacteria by recruitment and activation of macrophages. Li et al. (2013) demonstrated that *P. gingivalis* is able to reduce the expression of MIF mRNA in deep-pocket tissues (Li et al., 2013). Furthermore, *in vitro* experiments demonstrated that treatment of macrophages with the lysine-specific gingipain Kgp impaired its migration to apoptotic neutrophils and reduced the anti-inflammatory effect of apoptotic cells, resulting a rapid inflammatory response, leading the authors to suggest that *P. gingivalis* promotes chronic inflammation by a gingipain-mediated defect in apoptotic cell clearance and resolution of tissue restoration (Castro et al., 2017).

Following infection of epithelial cells and fibroblasts, *P. gingivalis* can also indirectly modulate immune cell functions. *In vitro* experiments showed that *P. gingivalis* infection of oral epithelial cells inhibits neutrophil migration (Madianos et al., 1997). Also, exposure of live *P. gingivalis* strain W83 to fibroblasts from periodontal ligaments *in vitro* induced a reduction in expression of macrophage colony-stimulating factor (Scheres et al., 2009). Those findings demonstrated that immune cell functions are also indirectly influenced by *P. gingivalis* infection, with bacterial secreted factors potentially a part of this complex system.

Overviews regarding the interactions of various other oral pathogenic bacteria with eukaryotic animal cells and cells from human sources have been presented (Feng and Weinberg, 2006; Kebschull and Papapanou, 2011). In general, *P. gingivalis* modifies antimicrobial host response and causes an imbalance in immune responses, leading to prolongation of inflammatory status and continuous damage against periodontal tissues. General bacterial burden, variability of colonizing species, oral hygiene, and other individual risk factors have further impact on host immune response and the subsequent outcome of periodontal disease.

## Potential Association of PD With Systemic Disease

Specific oral pathobionts influence related systemic diseases, such as atherosclerosis, infective endocarditis, diabetes, adverse pregnancy outcome, respiratory diseases, and RA, with various hypotheses based on epidemiological and experimental data presented. First, these systemic diseases and PD share common confounding factors, including lifestyle and/or genetic predisposition, indicating the importance of common host backgrounds. In addition, oral dysbiosis may cause autoimmunity via immune response against oral microbiota and subsequent molecular mimicry/autoantibody generation, as reported in cases of RA, in which T cell subsets are shaped toward pro-inflammatory cytokine-producing cells that drive autoimmunity development (Hooper et al., 2012). Since improved prognosis of patients with systemic diseases, such as coronary heart disease and RA, has been demonstrated following treatment for PD, a periodontal immune response and/or plaque bacteria provide a link for the mutual relationship between PD and those diseases (Montebugnoli et al., 2005; Al-Katma et al., 2007; Ortiz et al., 2009). In the following sections, possible relevance to the etiologies of PD and RA is discussed.

## Association of PD With RA

A reciprocal relationship between PD and RA has been reported (de Pablo et al., 2009; Koziel et al., 2014), and is also implied by the fact that early Assyrians 2,500 years prior treated rheumatism by tooth extraction. Even though no reliable records regarding the effect of that treatment exist, the concept of a mutual relationship of chronic joint disease with PD has been noted. However, the exact molecular and cellular mechanisms linking PD and RA are only slowly being unraveled. Based on a review of recent literature, reports supporting the various scenarios mentioned above are introduced here.

### Common Predisposing Factors of PD and RA

The first factors linking RA and PD include lifestyle and genetic predisposition as common confounders. Indeed, smoking and aging have been identified as risk factors for both of those diseases (Eriksson et al., 2016). As for a common genetic predisposition, data presented thus far remain inconclusive. While the strongest association for RA has been found among alleles of the HLA-DRB1^∗^04 and ^∗^01 haplotype groups, which carry a shared epitope, there is no significant association between HLA class II antigens and PD (Stein et al., 2008). Findings of an epidemiological study indicated that the hypomethylated status of a single CpG in the IL-6 promoter region plays a role in pathogenesis of RA and PD (Ishida et al., 2012). In an investigation of cases with aggressive PD, a large candidate-gene association study found no definite evidence for a genetic link of PD with RA, while their results suggested that IRF5 and PRDM1 are shared susceptibility factors, both of which are involved in interferon-β signaling, and also associated with systemic lupus erythematosus and inflammatory bowel disease (Schaefer et al., 2014).

### Oral Bacteria-Mediated Autoimmunity Links PD and RA

The second scenario, which has gained enormous attention, is oral dysbiosis as a prerequisite for pathogenic autoimmunity that leads to the onset of RA. Notably, the presence of *P. gingivalis* in PD lesions has been indicated as a link between both chronic inflammatory diseases (Rosenstein et al., 2004; Mikuls et al., 2009, 2014; Bartold et al., 2010). It was initially speculated that bacterial cell and/or toxin/metabolic byproducts can enter the systemic circulation from a clinically asymptomatic localized lesion containing pathogenic bacteria and spread to discrete anatomical sites, thereby initiating disease (Kumar, 2017). However, neither oral live bacteria nor their toxin/metabolic byproducts have been detected in the focus of the rheumatoid joint, though *P. gingivalis* DNA has been noted in synovial fluid (Reichert et al., 2013). On the other hand, immunological sequelae associated with oral pathobiont infection have gained considerable attention. These are formations of antibodies against citrullinated peptide antigens (ACPA) that precede the development of RA (Schellekens et al., 1998) and a molecular mimicry of bacterial proteins against host proteins, both of which raise autoantibodies.

Development of RA is attributable to production of ACPAs, the presence of which serves as a potent diagnostic marker for RA (Schellekens et al., 2000). Formation of ACPAs prior to RA onset has been the focus of intense research for the past two decades. While citrullination itself is a physiological process, the formation of antibodies against citrullinated peptide antigens is highly specific for RA (Schellekens et al., 1998). However, this specificity remains enigmatic, and whether these antibodies play an active role in the disease process or simply reflect an ongoing immune response has not been fully elucidated. On the other hand, the finding that citrullinated proteins accumulate in the joint provides a basis for interpreting the pathological condition in patients with RA. The pathology can be characterized as dysregulated citrullination, followed by release of neo-epitopes that breach immunological tolerance and trigger autoantibody formation.

### Biological Significance of Citrullination in RA Onset

Citrullination is mediated by peptidylarginine deaminase (PAD) enzymes. In humans, there are five different isotypes, PAD1-4, and PAD6, which exhibit a roughly 50–55% sequence similarity, and show distinct distributions in cells and tissues (Vossenaar et al., 2003; Zhang et al., 2004; Bicker and Thompson, 2013). Citrullination is the post-translational hydrolytic conversion of peptidyl-arginine into peptidyl-citrulline via deamination, a process that renders a reduction in the net positive charges of a given protein, thereby leading to increased hydrophobicity, protein unfolding, and altered intra- and inter-molecular interactions (Darrah et al., 2013). Physiologically, citrullination impacts gene regulation, terminal differentiation, and apoptosis, thus dysregulated citrullination is associated with numerous disorders, including autoimmune and neurodegenerative diseases (Witalison et al., 2015). PAD activity under a physiological condition is regulated by calcium concentration and a reducing environment (Arita et al., 2004). While full PAD activity *in vitro* requires millimolar amounts of calcium ion, intracellular nanomolar concentrations are likely to limit aberrant citrullination. Likewise, the oxidizing nature of the extracellular environment may provide protection from aberrant extracellular citrullination by PADs that may leak from activated or dying cells (Darrah et al., 2013). Of note, the citrullinome in RA is comprised of cytoplasmic and extracellular proteins, suggesting that both compartments are prone to dysregulated PAD activity. The major cell type for intracellular protein citrullination in the RA joint is represented by neutrophils, which are also the major source for soluble PAD2 and PAD4 released into synovial fluid (Romero et al., 2013; Spengler et al., 2015; Konig and Andrade, 2016). An important question then is what triggers hyper-citrullination in neutrophils? Among the various stimuli that trigger neutrophil activation and death, pore forming and membranolytic pathways that involve perforin and the complement membrane attack complex have been shown to induce intracellular calcium fluxes, a transient rise in intracellular calcium concentration and subsequent intracellular hyper-citrullination (Romero et al., 2013). Interestingly, the ability to provoke calcium influx-induced hyper-citrullination in neutrophils is definitely possessed by pore-forming immune mechanisms of the host, though that is also shared by bacterial calcium ionophores and pore-forming toxins (Konig et al., 2016). Due to limited conditions required for enzymatic activity, robust extracellular citrullination can only be maintained by a constant release of soluble PADs from dying cells and the presence of autoantibodies against PADs. Neutrophil NETosis and necrosis, as well as autophagy also contribute to extracellular hyper-citrullination via release of transiently active PAD enzymes (Spengler et al., 2015). Recently, it was shown that the presence of PAD3/PAD4 cross-reactive autoantibodies, which cause a decrease in calcium concentration required for catalysis, is associated with most erosive disease courses (Darrah et al., 2013; Navarro-Millán et al., 2016). As a consequence of hyper-citrullination, generation of neo-epitopes induced by changes in protein antigenicity might raise autoantibodies. Autoantibodies existing in synovial fluid opsonize target antigens and trigger a complement cascade, thus maintaining the vicious cycles of auto-inflammation and hyper-citrullination.

### Potential Involvement of Oral Pathobionts in ACP Generation

Bacterial toxins and enzymes have been reported to induce citrullination of host proteins and release of PADs. The pore-forming leukotoxin produced by Gram-negative *Aggregatibacter actinomycetemcomitans* kills human neutrophils and induces hyper-citrullination in neutrophils, thus contributing to dysregulated citrullination (Konig et al., 2016). Likewise, as a putative link between PD and RA, the pathobiont *P. gingivalis* expresses a prokaryotic PAD (PPAD), which is thus far unique among microorganisms. In contrast to human PADs, PPAD does not require calcium ion for its activity of citrullination of C-terminal arginine residues (Rodriguez et al., 2009; McGraw et al., 1999). Furthermore, while PADs are unable to catalyze free L-citrulline, PPAD can citrullinate both free and peptide-bound arginine (Abdullah et al., 2013). A cellular PPAD with an approximate size of 75–85 kDa and a secreted PPAD sized 47 kDa have been described (Konig et al., 2014). PPAD is extracellularly secreted or located in the outer membrane of *P. gingivalis* together with virulence factors, arginine-specific gingipains RgpA and RgpB, which cleave the carboxyl group of arginine residue in their own target proteins. The cleaved products exposing arginine residue at the carboxyl terminus are prone to rapid citrullination by PPAD (Wegner et al., 2010; Maresz et al., 2013).

Prokaryotic PAD also citrullinates human proteins, such as fibrin, vimentin, epidermal growth factor (EGF), fibrinogen, and α-enolase (McGraw et al., 1999; Mangat et al., 2010; Wegner et al., 2010; Montgomery et al., 2016). Therefore, host proteins modified by PPAD may function as antigens that induce generation of ACPAs (Vossenaar and van Venrooij, 2004; Moscarello et al., 2007; Nesse et al., 2012). As a consequence, citrullination of EGF results in defects in cell-cycle modulation. Since EGF activates cell proliferation, migration, repair, and regeneration of gingival epithelial cells, its citrullination hampers regeneration of damaged tissue. As a result, modification of human proteins mediated by PPAD likely induces a biological shift in the local environment (Pyrc et al., 2013). Also, PPAD may be important for interactions of *P. gingivalis* with eukaryotic cells, including neutrophils, macrophages, and epithelial cells (Quirke et al., 2014). Additionally, it has been shown that monocytes and macrophages exposed to viable *P. gingivalis* had increased extracellular citrullination levels, while the endogenous PAD level is not affected (Marchant et al., 2013). Bielecka et al. (2014) also showed that PPAD citrullinates the C-terminal arginine residue of the chemoattractant complement factor C5a, resulting in decreased chemotaxis of human neutrophils and release of pro-inflammatory cytokines from immune cells (Bielecka et al., 2014). However, the extent to which PPAD enzymatic activity affects the functions of oral host cells, including immune cells, remains elusive. As a consequence of citrullination of bacterial proteins, auto-citrullination of PPAD and antibodies against gingipains can be detected in both PD patients and healthy individuals. On the other hand, antibodies against citrullinated PPAD are specific to RA, suggesting that citrullinated PPAD is a member of early induced proteins that contribute to ACPA generation.

In the context of mechanisms that induce antibody cross-reactivity, *P. gingivalis* can citrullinate its own α-enolase, which shares an 82% sequence homology with human α-enolase, a finding that provides a convincing argument in terms of epitope spreading and molecular mimicry (Lundberg et al., 2008). Therefore, PD patients colonized with *P. gingivalis* might produce antibodies against citrullinated bacterial proteins homologous to human proteins and this molecular mimicry of the antigen may elicit an immune response to human tissues. Similarly, PPAD citrullination of host proteins allows for neo-epitope formation that triggers autoimmune responses.

### *In Vivo* Evaluations of PPAD Functions Connecting PD and RA

Due to diverse factors that influence PD and RA, animal models have been utilized to detect a causal relationship between those diseases. Kinloch et al. (2011) demonstrated that citrullinated enolase induces experimental arthritis, and showed that enolase citrullinated by human PAD or *P. gingivalis* PPAD induces autoantibody production in DR4-IE-transgenic mice (Kinloch et al., 2011). Utilizing a collagen-induced arthritis (CIA) mouse model, Maresz et al. (2013) demonstrated that the ability of *P. gingivalis* strain W83 to augment CIA was dependent on PPAD activity. Moreover, infection with the wild-type strain, but not its PPAD-null mutant, induced elevated levels of autoantibodies to collagen type II (Maresz et al., 2013). These *in vivo* results emphasize the importance of PPAD as a potential virulence factor of *P. gingivalis* and a key component connecting PD and RA.

### Relevance of ACPAs in Pathogenesis of RA

Recent findings have demonstrated that osteoclasts express PAD enzymes at all stages of their development, while detectable citrullinated proteins have also been detected on their cell surface (Harre et al., 2012; Krishnamurthy et al., 2016). ACPAs can therefore bind to osteoclast precursors and induce expression of IL-8, which acts as an autocrine growth factor and drives differentiation into mature bone-resorbing osteoclasts (Kopesky et al., 2014). While those findings account for bone-resorption mechanisms to some extent, they are clearly not sufficient to induce chronic synovial inflammation (Catrina et al., 2017). Indeed, animal models have shown that a single ACPA administration does not induce arthritis. However, if mild synovial inflammation already exists, severe joint disease reminiscent of human RA has been found to develop in the presence of ACPAs (Sohn et al., 2015). These findings suggest that ACPAs are important but not alone sufficient for inducing chronic inflammation, though they apparently play an active role in the disease processes leading to RA.

## Summary

Oral pathobionts are constituents of a complex ecosystem and provide a mutually trophic metabolism together in a state of equilibrium with host factors and the immune system. Dental biofilms in each anatomical site are characterized by a distinct composition of bacterial species. A continuous existence of periodontal biofilm exacerbates host inflammatory response and drives a shift in the periodontal microbiome, which leads to onset of PD. As a red complex member, *P. gingivalis* affects the functions of various host cells and manipulates antimicrobial host response, thereby posing dysbiosis and prolongation of an inflammatory status along with periodontal tissue damage. Furthermore, recent findings indicate that particular periodontal pathobionts, such as *P. gingivalis*, have an exacerbating role for generation of ACPAs, which has been confirmed by epidemiological data showing an interrelationship between PD and RA. ACPAs activate immune response, including complement activation, and thus facilitate local hyper-citrullination, while they also activate osteoclasts to absorb bone and provide the basis for RA development. Imbalances in the oral microbiome shape the pro-inflammatory axis of the cytokine network, which offers a broad framework to comprehend the pathogeneses of autoimmunity and chronic inflammatory diseases (Hooper et al., 2012). However, the exact mechanisms by which oral pathobionts have effects on both the cytokine network and autoimmunity remain obscure. Further research is needed to evaluate the involvement of genetic/environmental susceptibility factors, citrullination, and cytokine networks in the reciprocal relationship of PD and RA. As shown in this review, it is currently not possible to finally conclude if oral pathobionts like *P. gingivalis* can simply be classified as bystander microbiota or if they are disease initiators, with the consequence that perhaps early prophylactic treatment could prevent systemic and chronic diseases, such as atherosclerosis, infective endocarditis, diabetes, adverse pregnancy outcome, respiratory diseases, and RA.

## Author Contributions

All authors conceived the concept for this review article and participated in writing the manuscript. Each equally contributed to reading, editing, and reviewing the manuscript.

## Conflict of Interest Statement

The authors declare that the research was conducted in the absence of any commercial or financial relationships that could be construed as a potential conflict of interest.
